# Flexible and sewable electrode based on Ni-Co@PANI-salphen composite-coated on textiles for wearable supercapacitor

**DOI:** 10.1038/s41598-023-47067-y

**Published:** 2023-11-13

**Authors:** Touba Rezaee Adriyani, Ali A. Ensafi, B. Rezaei

**Affiliations:** 1 Department of Chemistry, Isfahan University of Technology, Isfahan, 84156-83111 Iran; 2 Department of Chemistry & Biochemistry, University of Arkansas, Fayetteville, AR 72701 USA

**Keywords:** Chemistry, Energy science and technology, Materials science, Nanoscience and technology

## Abstract

Flexible electrodes with high deformability and energy density are critical for electronic textiles. The key factor for achieving high-performance supercapacitors with superior power and energy density is the evaluation of materials that exhibit exceptional capacitive performance. Herein, we have prepared Ni-Co nanoparticles at the surface of polyaniline-salphen (Ni-Co@PS). Then, followed by casting Ni-Co@PS on a conductive carbon cloth (CC) as a substrate through a facile in-situ polymerization strategy. The morphologies of Ni-Co@PS composite were characterized by different methods such as FE-SEM, XPS, XRD, BET, and electrochemical methods. This nanocomposite showed high tolerability and a large surface area with excellent behavior as a new nanomaterial for supercapacitor application. Thus, the optimum composite designed with a metal ratio (nickel–cobalt 3:1 w/w) satisfactorily possesses a specific capacitance of up to 549.994 C g^−1^ (1447.2 F g^−1^) under 0.5 A g^−1^ and long-term cyclic stability featuring capacity retention of 95.9% after 5000 cycles at a current density of 9.0 A g^−1^. The Ni-Co@PS-CC, is a material with great potential as an electrode in asymmetric wearable supercapacitor (AWSC) apparatus, demonstrating a remarkable specific capacity of 70.01, and accompanied by an energy density of 23.46 Wh k g^−1^ at a power density of 800 W k g^−1^.

## Introduction

Wearable electronic textiles (E-textiles) are predicted to be as flexible, breathable, and comfortable as regular clothing when worn directly on the body. Intelligent E-textiles have great potential for addressing urgent global issues by providing solutions in the areas of healthy aging, patient monitoring, emergency response, and environmental monitoring^1–4^. These smart devices have become a noticeable technological trend undergoing accelerated expansion Internet of Things (IoT) development. The E-textiles devices should have a high density of active and passive components that are seamlessly integrated and self-powered (for example, sensors, actuators, displays, and microcontrollers (analysis/transfer information)). Nowadays, the power supply in E-textiles devices is dominated by electrochemical-based energy storage systems. These devices should have a compact volume, sewable, lightweight, and biocompatible performance^5–7^.

The electrochemical-based energy storage system is highly esteemed by academics for its implicity and extensive applicability in industrial applications. Electrochemical capacitors (or supercapacitors (SCs)) and Lithium-ion batteries (LIBs) are two significant subcategories of the electrochemical-based energy storage system. These applications encompass a wide variety of uses, including providing energy for portable as well as for wearable devices. The remarkable performance of this supercapacitor exemplifies a pragmatic instance of a self-sustaining power system that is conveniently transportable and possesses extensive potential for the future development of portable and wearable electronics^8^.

The other notable point in these energy storage devices contain safety electrolytes that pose a decreased safety risk in wearable systems and necessitate special packaging to prevent leakage of contents from the instrument^9^.

Attempts are being made to integrate these wearable supercapacitors, as power sources, with various wearable and flexible electronic devices to develop the concept of self-powered smart electronic devices, but the underlying process is not as straightforward as it seems. Supercapacitors can be categorized into two groups based on their storage mechanism: pseudo-capacitors and electrochemical double-layer capacitors (EDLCs). The efficiency of an EDLC is related to the properties of the electrode material such as nature and electrochemical activity. Appropriate pore distribution, large porosity, and high specific surface area are required in the electrode fabrication process^10–13^. In this regard, several types of materials such as conductive polymers (polyaniline (PANI) and polypyrrole (PPy)) and polymeric transition metal complexes have been adopted to fabricate supercapacitors^12,14,15^. PANI has garnered considerable interest for commercial applications due to its unique advantages, including one-step preparation, economical price, excellent stability, and high intrinsic conductivity due to their π-conjugated electronic systems. Additionally, biocompatibility refers to the capacity of materials to coexist with living organisms and tissues without causing harm. As per the ISO 10993 guidelines, PANI exhibits biocompatible characteristics concerning dermal irritation and sensitization^16^. The pseudocapacitive behavior of PANI is characterized by phenomena like shrinkage, swelling, and cracking occurring during the process of doping and de-doping of charged ions. These phenomena contribute to the limited cyclic stability observed in PANI-based systems. One of the primary drawbacks associated with PANI pertains to its volume expansion during the charging and discharging process, which subsequently results in a diminished cycle life^17^. Additionally, PANI exhibits low conductivity while in the de-doped form^18^. One plausible strategy for augmenting the conductivity and stability of PANI involves its integration with other conductive materials, such as reduced/graphene oxide, and metal-based complex^19^. Customizing the properties of conductive PANI by hybridizing pseudocapacitive materials has been demonstrated in scores of research^20,21^.

Polymeric transition metal complexes such as salen and salphen (SOF) are hopeful pseudocapacitive materials for energy storage. These materials are fabricated by Schiff base ligands that synthesized from salicylaldehyde and derivatives. However, many researchers have described the synthesis of SOF with π-conjugated ligands between the coordination sites and metals^22,23^. SOFs have both carbon sources and metal, as well as excellent properties, making them ideal materials for electrode functional nano-material^24,25^. The vital point in wearable supercapacitor devices is the biocompatibility of all outer and inner materials used in the final assembly^26^. The cytotoxicity of composites is contingent upon various factors such as the chemical composition, size, and shape of the materials^16,27^. In the previous literature cytotoxic effect of several polymeric transition metal complexes (such as salen) has been demonstrated^25,26,28^. As indicated in the aforementioned literature, salphen exhibits favorable biocompatibility properties. Transition metals often have lower electrical conductivity in comparison to carbon-based materials within a range of other supercapacitor materials. Likewise, in the case of carbon-based materials, it is frequently seen that they exhibit regular forms and a same pore size. Materials-based transition metals are a prominent class of materials for supercapacitors, surpassing the aforementioned materials in importance^29^. The binary system of nickel and cobalt has the potential to exhibit a remarkably high capacitance, owing to the abundance of Faradaic redox reactions that stem from the rich valence states of the constituent Ni and Co metals. This results in an exceptional capacitance performance of the system. The comparable atomic dimensions of nickel and cobalt hold significance in the synthesis of the bimetallic salphen complex. The commensurate atomic sizes of Ni and Co are widely acknowledged, rNi measuring 1.246 Å and rCo measuring at 1.253 Å^30,31^. The incorporation of transition metals (ratio and type) in salphen complex and composite, specifically with regard to their redox properties, synthesis pathway, and energy storage mechanisms, numerous investigations and research endeavors might be conducted on this topic. There is a Comprehensive evaluation of the study on the impact of the ratio and type of metals in the salphen@polyaniline composite on wearable pseudocapacitive energy storage.

In the present work, we report successfully designed and synthesized metal-salphen complex via a simple emulsion polymerization within a porous PANI. On the other hand, the salphen complex avoids the dissolving of the metals in the electrolyte causing improvement in the performance of the supercapacitor. The control of the synthesis parameters such as metals salt concentration and PANI ratio allowed the optimization of the Ni-Co at the surface of polyaniline-salphen (Ni-Co@PS) composite for excellent supercapacitor performance. This paper also demonstrated the performance of supercapacitor electrodes for wearable systems.

## Results and discussion

### Characterization of the electrode materials

**Figure 1:**
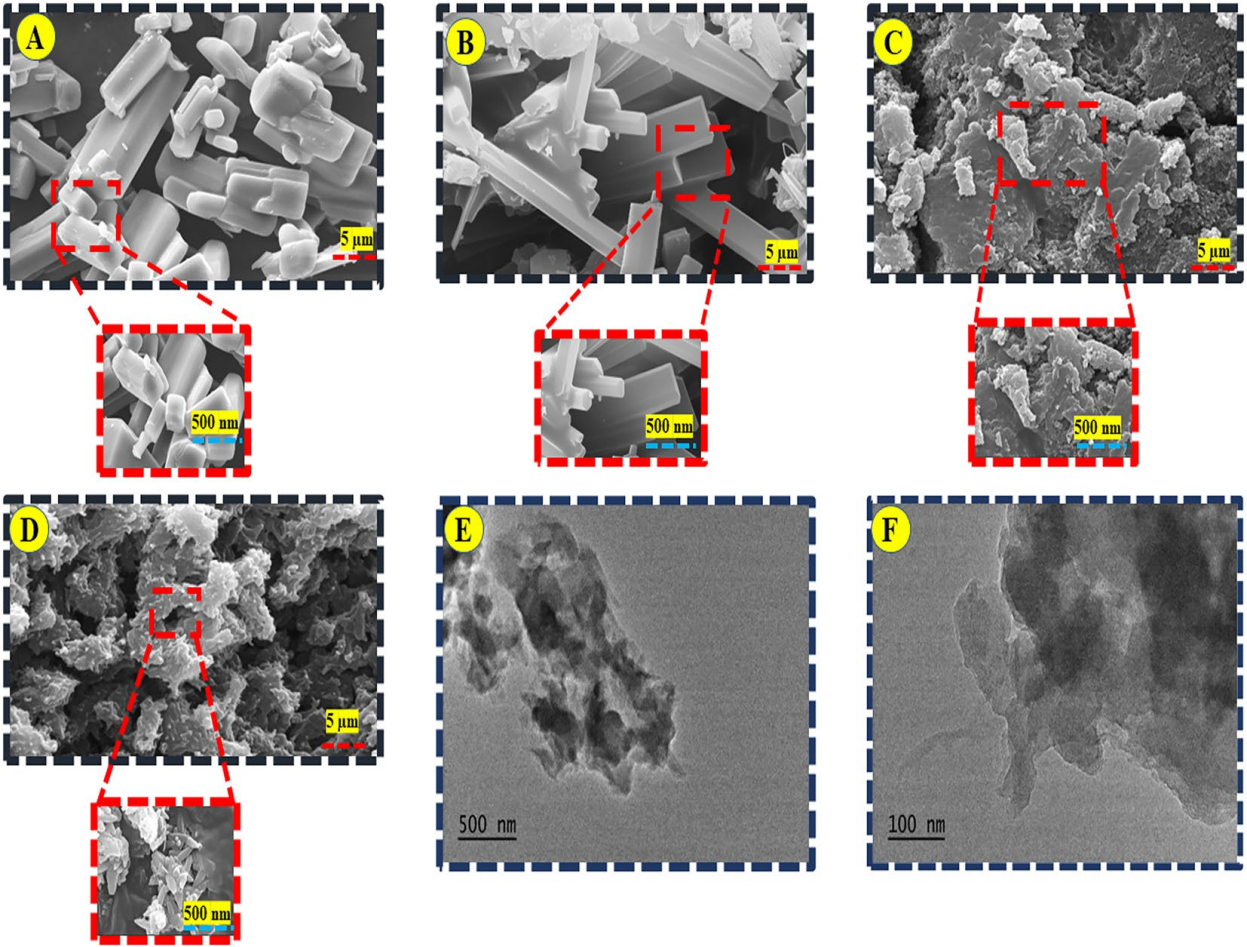
FE-SEM images of (**A**) Salphen, (**B**) Ni-Co(1:3)@S, (**C**) PANI, and (**D**) Ni-Co(3:1)@PS composite. (**E**) and (**F**) TEM image of Ni-Co(3:1)@PS composite.

The surface morphologies of the prepared materials were investigated by FE-SEM technique. Figure 1A–D shows the FESEM image of the salphen ligand, Ni-Co@S complex, PANI, and Ni-Co(3:1)@PS composite, respectively. It can be observed that both complex and metal complex have the shape of hexagonal and that images show the particles have a closely packed arrangement structure (Fig. 1A and B). The morphology of the synthesized salphen ligand, Ni-Co@S complex was similar to the FE-SEM images reported previously^32^. This aggregation is anticipated to result from the dipolar antiferromagnetic interactions between Co and Ni ions at both complex sites. The FE-SEM image of PANI appears a random interconnected network and flat plate-like structure (Fig. 1C)^33^. As shown in Fig. 3D, the morphology of the synthesis of Ni-Co(3:1)@PS composite was like the metal complex image, which is dispersed onto plate-like PANI and have smaller particles. However, under conditions of nearly identical in-situ polymerization, there are few morphological distinctions for salphen complex (Ni-Co) in the composite. The small size of the salphen complex (Ni-Co) leads to the H bond, π–π, and π stacking interaction between the π-conjugated metal complex and PANI^34^. Additionally, TEM images of Ni-Co(3:1)@PS composite showed the metal salphen complex possesses a porous and wrinkled wavy structure, which is covered with a PANI layer as shown in Fig. 1E, F. This observed result completely matches with the FE-SEM images.

**Figure 2:**
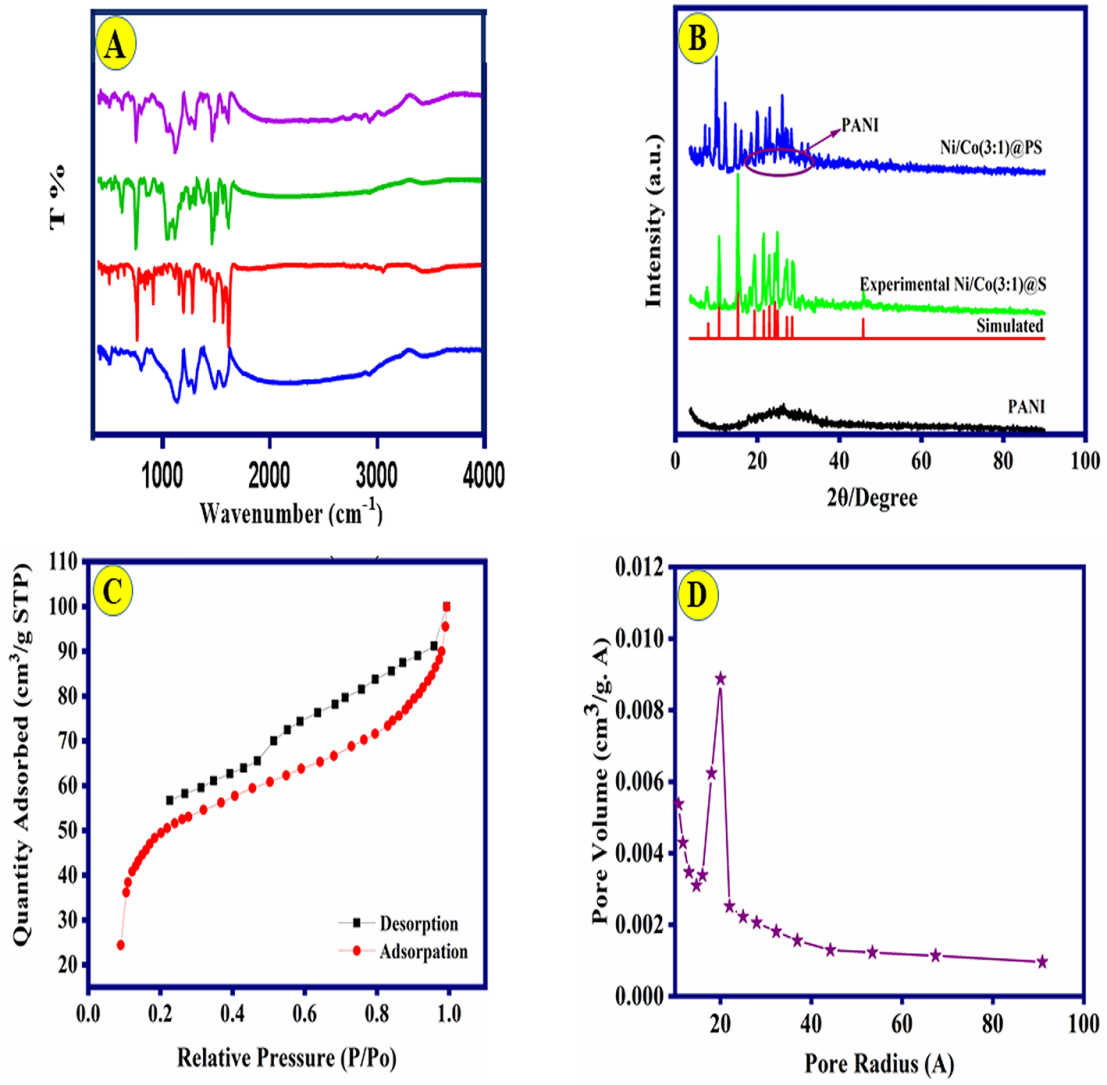
(**B**) XRD patterns of PANI, Ni-Co(3:1)@S, and Ni-Co(3:1)@PS composite; (**A**) FT-IR spectra of PANI, salphen, and Ni-Co(1:3)@S, Ni-Co(3:1)@PS composite (as-prepared samples). (**C**, **D**) N_2_ adsorption/desorption curve and pore dimension distribution for Ni-Co(3:1)@PS composite.

To further investigate the elemental composition and distribution of the electrode material, EDAX and MAP pattern were obtained. Figure S1C illustrates the pattern of the PANI, where only elements such as N and C are observed in the graph and the results correspond to the literature^27^. Moreover, the distribution and elemental composition of the complex and metal complex were displayed in Fig. S1A, B. From the EDAX results, it can be verified Ni and Co in the metal complex. The EDAX and map pattern of the final composite shows the signal of PANI and complex in the pattern related to the Ni-Co(3:1)@PS composite. This result indicates the incorporation of the metal complex into the PANI matrix.

Figure 2A demonstrated the FT-IR patterns of PANI, Ni-Co@S complex, and Ni-Co(3:1)@PS composite, respectively. The IR spectral data of the electrode materials are given in TableS3^26,27,32,35^. XRD spectra were obtained, and they were compared to investigate the crystalline compounds and phase identification of the supercapacitor’s material. Figure 2B compares the XRD spectra analysis of the electrode materials during the synthesis steps. The spectra of PANI exhibit a broad peak, indicating the amorphous characteristics of PANI. This observation is also mentioned in research articles^27,32,36^. The pure PANI material exhibits distinct diffraction peaks at 25.14°, which corresponds to the crystal plane (2 0 0). This observation is quite consistent with the ICCD No. 09-0836. Polyanilines exhibit a semi-crystalline structure and are composed of two distinct phases. The crystallite area refers to the phase in which the polymer chains are arranged parallel and closely packed. On the other hand, the amorphous region is characterized by the absence of order and parallel alignment among the chains^37^. On the other hand, when the polymer's chain rapid growth resulted in a lack of order in the alignment of polymer chains and the formation of an amorphous polymer structure^38^. The occurrence of crystallinity in the Ni/Co (3:1)@S complexes and final composite is due to the intrinsic crystalline characteristics of the metallic compounds. As can be seen in the pure PANI pattern, the main diffraction peaks at position 25.14° which corresponds to (2 0 0), this result is well consistent with the ICCD No. 09-0836. The presence of crystallinity in the Ni/Co(3:1)@S complexes and composite can be attributed to the intrinsic crystalline properties of the metallic compounds. The crystal structure of this shift base ligand has previously been documented by Reyes-Gutiérrez et al.^39^. The reference code for this structure in the Cambridge Structure Database (CSD): EKEYEA is available. The diffraction peaks were clearly observed at the 2θ positions of 8.12°, 10.51°, 15.25°, 19.4°, 21.55°, 22.95°, 24.4°, 24.51°, 27.20°, 28.2°, and 45.85°, which closely match the values reported in the literature. This confirms that the synthesis of this final composite was effective. The diffraction peaks was apparent at the 2θ position of 8.12°, 10.51°, 15.25°, 19.4°, 21.55°, 22.95°, 24.4°, 24.51°, 27.20°, 28.2°, and 45.85° which fits well with the literature, confirming the successful synthesis of this compound^26,40–42^. The most important diffraction peak (I_max_) was observed at 15.52° (2θ) with a d-spacing value of 4.818. The introduction of amorphous components such as PANI in the final composite results in a decrease in crystallinity, as indicated by the observed decrease in diffraction peak sharpness. The loss in crystallinity that happens as a consequence of the inclusion of amorphous components (PANI) into the Ni-Co@PS composite, is evidenced by the fact that the sharpness of the diffraction peak is also decreased as a consequence of this incorporation. This demonstrates that the incorporation of amorphous components leads to a reduction in the crystallinity of the Ni-Co@PS composite. The findings derived from the XRD analysis exhibit substantial concurrence with the outcomes of the FESEM analysis. In addition, the residual Co and Ni ions contents in Ni-Co(3:1)@PS composite were detected to be at 13 and 29 mg g^−1^, using inductively ICP-AES. Figure 2C presents the results of the BET analysis conducted on the produced composite. The Ni-Co@PS composite exhibits type IV isotherms in accordance with the International Union of Pure and Applied Chemistry (IUPAC) categorization of pores. The BET surface area of the Ni-Co@PS composite was measured to be 166 m^2^ g^−1^, while the total pore volume was determined to be 18.62 cm^3^ g^−1^. The observed large surface area and pore volume of the Ni-Co@PS composite sample can potentially be attributed to the prevalence of hexagonal-like structures characterized by favorable microporosity. This structural characteristic contributes to higher capabilities in terms of electrolytic charge storage and ion transport, ultimately leading to improved supercapacitor performance.

**Figure 3:**
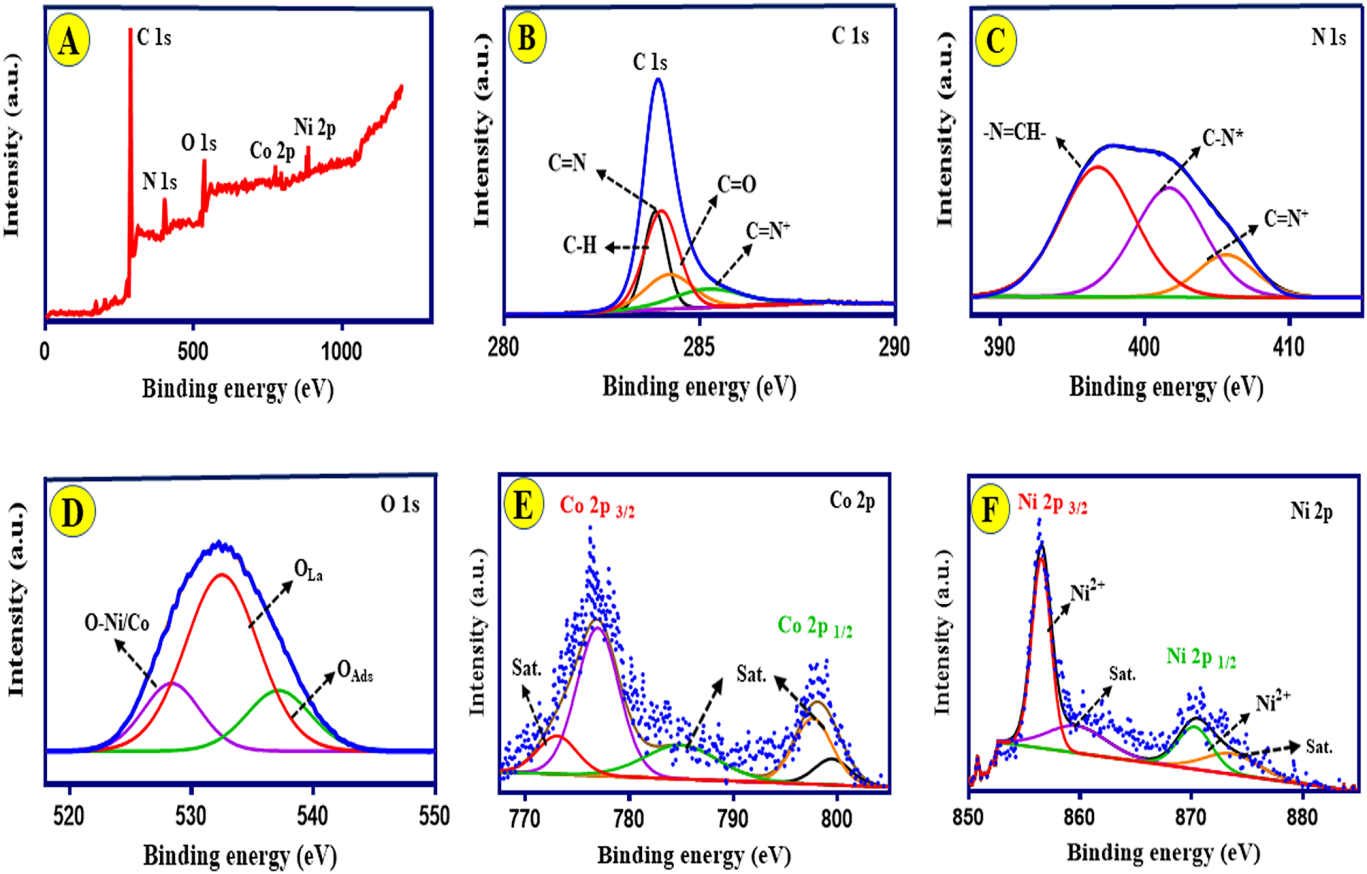
(**A**) Total survey scan XPS spectrum of Ni-Co(3:1)@PS composite; (**B–F**) Core-level XPS spectra of C 1s, N 1s, O 1s, Co 2P, and Ni 2p, respectively.

To better understand the energy storage mechanism and chemical state of the Ni-Co(3:1)@PS, surface analysis of the composite was carried out using XPS method. This data about the fitting after in-situ polymerization was summarized in Fig. 3A–F. Figure 3A displays the survey graph calibrated by C 1s spectrum at 284.6 eV. The Ni 2p, Co 2p, O 1s, N 1s, and C 1s photoemission peaks in the survey graph are found for the Ni-Co(3:1)@PS composite. The observed photoemission peaks can be ascribed to the co-existence of metal-salphen complexes and PANI, which is the result according to the literature^43,44^. The investigation of the fabrication of composite and surface characterization required the examination of high-resolution XPS spectra.

The high-resolution C 1s exhibit four peaks at 283.90, 284.10, 284.17, and 286.01 eV, which correspond to (C−H, C=N, C=O, and C=N^+^), as shown in Fig. 3B. The N 1s (Fig. 3C) displays three peaks, the low energy peak assigned to the azomethine nitrogen group (−N=CH−) coordinated with the metal ion, while the middle and high energy peak corresponding to the π–π* interaction (C–N*) and protonated (imine = N^+^), respectively^45^. In the high-resolution spectrum (Fig. 3D), the oxygen core level spectrum shows three peaks that are exhibited and centered at 527, 532, and 537 eV, which can be corresponded to metal–oxygen bonding (Ni/Co–O), lattice oxygen (O_la_), and adsorbed oxygen (O_abs_), respectively. The confirmation of the presence of Co(II) or Co(III) oxide species is further evidenced by the observed binding energies of Co 2p3/2 and Co 2p1/2, which are located at 775.95 and 799.89 eV, respectively Fig. 3E^45^. The Co 2p spectra exhibit distinct shakeup satellite peaks, a finding that aligns with previously documented observations of CoOx^46,47^. Figure 3F apparent two separated and well-defined peaks at a binding energies of 855 and 871 eV, which can be assigned as Ni 2p_3/2_ and Ni 2p_1/2_, respectively. This observation suggests the presence of nickel in the Ni(II) oxidation state. The presence of Ni(II) ions lead to alterations in the electronic configurations of the composite, hence increasing its supercapacitor performance^48^. Figure 3E, F indicate the Ni and Co embedded into the salphen complex during the synthesis process. The satellite peaks are due to photoelectrons emitted at lower kinetic energies from Ni 2p_3/2_, Ni 2p_1/2_ and Co 2p_3/2_, and Co 2p_1/2_^43^_._

### Electrochemical performance test

**Figure 4:**
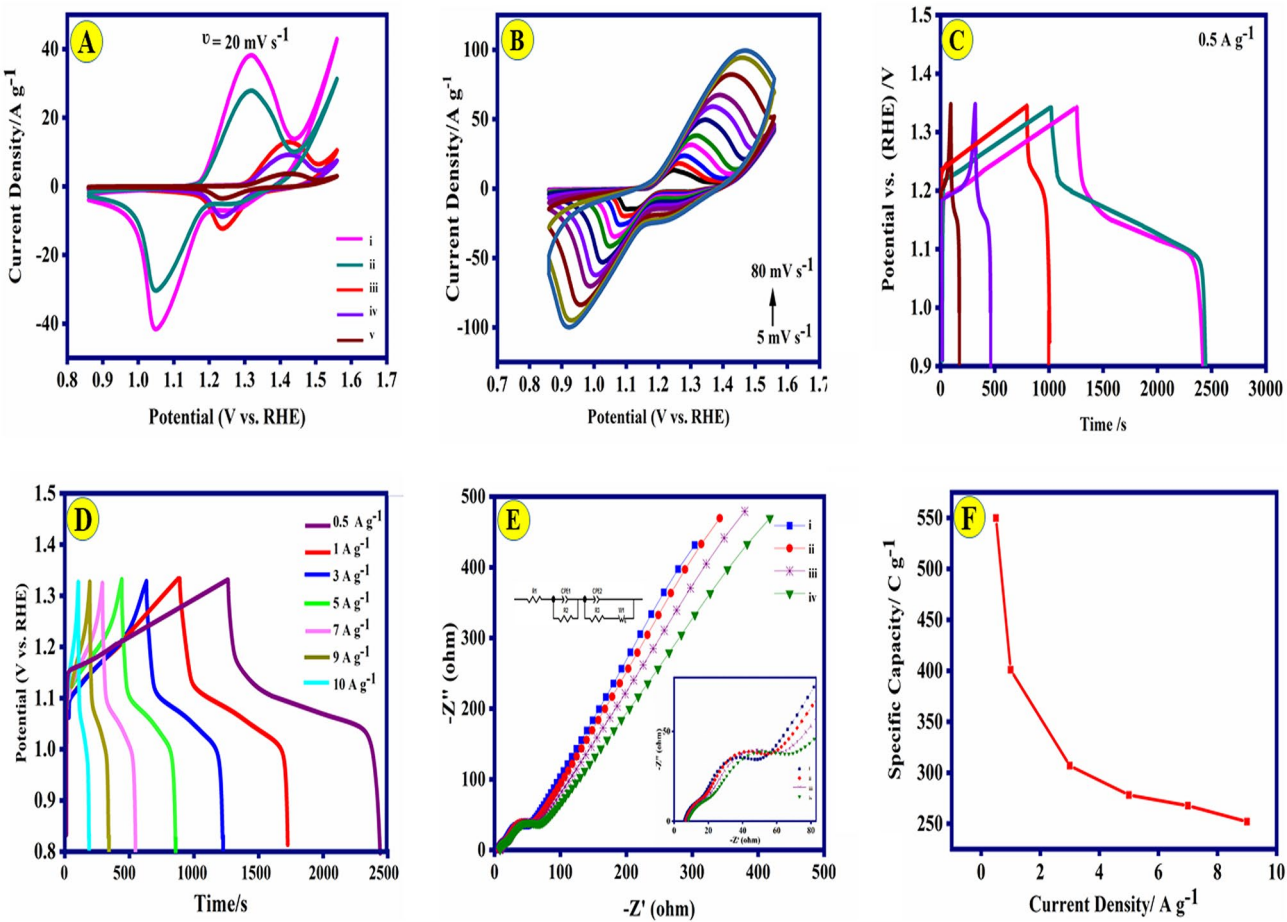
(**A**) Cyclic voltammetry of (i) Ni-Co(3:1)@PS-CC, (ii) Ni-Co(2:1)@PS-CC, (iii) Ni-Co(1:2)@PS-CC, (iv) Ni-Co(1:3)@PS-CC, and (v) PS electrodes (at the scan rate 20 mV s^−1^); (**B**) CV curves of the Ni-Co(3:1)@PS-CC at different scan rate (5–80 mV s^−1^); (**C**) Charge and discharge profiles of (i) Ni-Co(3:1)@PS-CC, (ii) Ni-Co(2:1)@PS-CC, (iii) Ni-Co(1:2)@PS-CC, (iv) Ni-Co(1:3)@PS-CC, and (v) PS electrodes at 0.5 A g^−1^; (**D**) GCD profiles of the Ni-Co(3:1)@PS-CC electrode at various current densities; (**E**) EIS spectrum of (i) Ni-Co(3:1)@PS-CC, (ii) Ni-Co(2:1)@PS-CC, (iii) Ni-Co(1:3)@PS-CC, and (iv) Ni-Co(1:2)@PS-CC with its equivalent circuit; (**F**) the Ni-Co(3:1)@PS-CC electrode capability at different current densities.

To assess the electrochemical efficacy of the as-fabricated composites, CV and GCD techniques were implemented in 1.0 M KOH solution. CV profiles of the various modified electrodes were recorded (Fig. 4A) at a constant scan rate of 20 mV s^−1^ over the potential range of 0.85–1.60 V (vs. RHE). The cyclic voltammograms for the composites indicated the quasi-reversible shape, which implies the contributions of both EDLC and pseudo-capacitance of the electrodes. The Ni-Co(3:1)@PS-CC material exhibits distinct characteristics, specifically the presence of redox peaks. The first anodic peak observed at a potential of 1.31 V (vs. RHE), while the cathodic peak displayed at a potential of 1.05 V. The redox peak that appears in the CV curve was observed attributed to the redox of Ni, Co, PANI, and salphen. By comparing the area of the CV curve, it was discovered that the capacitance of the CC-Ni-Co(3:1)@PS electrode with Ni-Co ratio (3/1) in the complex structure is significantly higher than another composite materials. According to the results obtained from the CV, increasing the ratio of the Ni to Co enhanced the capacitive properties. Figure 4B shows the CVs of Ni-Co(3:1)@PS-CC electrodes at different scan rates (5–80 mv s^−1^). Upon increasing the scan rate from 10 to 100 mV s^−1^, no significant alteration in the shape of the voltammogram was observed, indicating the Ni-Co(3:1)@PS-CC electrodes possess good electrochemical reversibility and fostering mass transfer in the KOH solution. As a result of the polarization effect occurring on the electrode surface, the peaks of the oxidation and reduction are shifted towards higher and lower potentials, respectively. This phenomenon leads to an expansion of the working potential windows. The results confirm that the current intensity of the redox peak is enhanced with increasing the scan rate.

Figure 4C illustrates the GCD for all materials electrode that conducted at a current density of 0.5 A g^−1^. The curved shape of the GCD plot serves as evidence for the presence of the pseudo capacitance mechanism and Faradaic reaction involving Ni^2+^/Ni^3+^ and Co^3+^/Co^4+^. These redox reactions, which are attributed to the redox of PANI, salphen and electrochemical transformations of M–OH (M=Ni and Co) in the presence of typical alkaline electrolytes, are expressed as follows:

1 $${\text{Co}}_{{3}} {\text{O}}_{{4}} + {\text{ OH}}^{ - } + {\text{H}}_{{2}} {\text{O}}{ \leftrightharpoons }{\text{3CoOOH }} + {\text{ e}}^{ - }$$

2 $${\text{CoOOH }} + {\text{ OH}}^{ - } \rightleftharpoons {\text{CoO}}_{{2}} + {\text{ H}}_{{2}} {\text{O }} + {\text{ e}}^{ - }$$

3 $${\text{Ni}}\left( {{\text{OH}}} \right)_{{2}} + {\text{ OH}}^{ - } { \leftrightharpoons }{\text{NiOOH }} + {\text{ H}}_{{2}} {\text{O }} + {\text{ e}}^{ - }$$

The symmetric and liner plot displays the ideal capacitive performance of the Ni-Co(3:1)@PS-CC electrode. This result agrees with the results of the CV curves. The electrochemical performance of electrodes can be calculated by using the equations shown in Table S4. The high capacity of Ni-Co(3:1)@PS-CC electrode as 549.94 C g^−1^ is obviously proved. The capacitive properties are optimized by the inclusion a of the metal’s ratio. The capacitance behavior of the results nanocomposites is directly proportional to the metal ratio present. At 0.5 A g^−1^, the areal capacitance of Ni-Co(3:1)@PS-CC (549.94 C g^−1^) is higher than Ni-Co(2:1)@PS-CC (374.34 C g^−1^), Ni-Co(1:2)@PS-CC (92.54 C g^−1^), and Ni-Co(1:3)@PS-CC (35.32 C g^−1^), respectively. Therefore, Ni-Co(3:1)@PS-CC was used to conduct further investigations. As can be seen in Fig. 4D, the specific capacity of Ni-Co(3:1)@PS-CC electrode was calculated as 549.94, 400.85, 306.85, 278.22, 267.66, 251.93 C g^−1^ at different current densities of 0.5, 1.0, 3.0, 5.0, 7.0, and 9.0 A g^−1^, respectively. Due to the insufficient association of redox-active materials with the electrolyte, increasing the current density reduces the specific capacitance. The ESI technique was utilized to examine the kinetic properties of composite materials. The Nyquist plots of different ratios of the metals in the composite were recorded in 1.0 M KOH solution at the frequency range of 10 Hz–100 MHz (Fig. 4E). The plots exhibit two quasi-semicircular patterns in the high-frequency range while displaying a linear pattern in the low-frequency range. The presence of PANI and metal-salophen gives rise to two distinct electron transfer models, which are observed as two quasi-semicircular patterns in the analysis of ESI. In addition, the Bode phase angle plot also confirms this finding that it is available in Fig. S2. It might be argued that the initial semicircle, characterized by its small dimensions and occurrence at significantly higher frequencies, is the same for all electrode materials, perhaps indicating the presence of PANI. The intersection of the arc of the quasi-semicircle with the axis within the high-frequency region corresponds to the parameter Rs, which signifies the resistance at the interface between the electrolyte and the electrode. One could assume that the resistance values (R_s_) are derived from the same point of origin and exhibit consistency across all electrodes. To do a comparative analysis of these electrodes, the second quasi-semicircular has been investigated. The observation of Fig. 4E reveals that the curve in the high-frequency region has a shape resembling that of a semicircle, with the diameter of said semicircle corresponding to the charge transfer resistance (R_ct_) of the active material. The charge transfer resistance decreases as the diameter of the semicircle decreases. According to the results obtained, it can be concluded the R_ct_ decreased from 44.85 Ω for Ni-Co(3:1)@PS-CC to 37.21 Ω for Ni-Co(2:1)@PS-CC, 31.52 Ω for Ni-Co(1:2)@PS-CC and 22.85 Ω for Ni-Co(1:3)@PS-CC. These results suggest that Ni-Co(3:1)@PS-CC has a fast charge transfer and better capacitance performance. The results indicate that Ni-Co(3:1)@PS-CC was able to maintain a specific capacitance of 45.8% with an increase in current density from 1.0 to 9 A g^−1^, (Fig. 4F).

**Figure 5:**
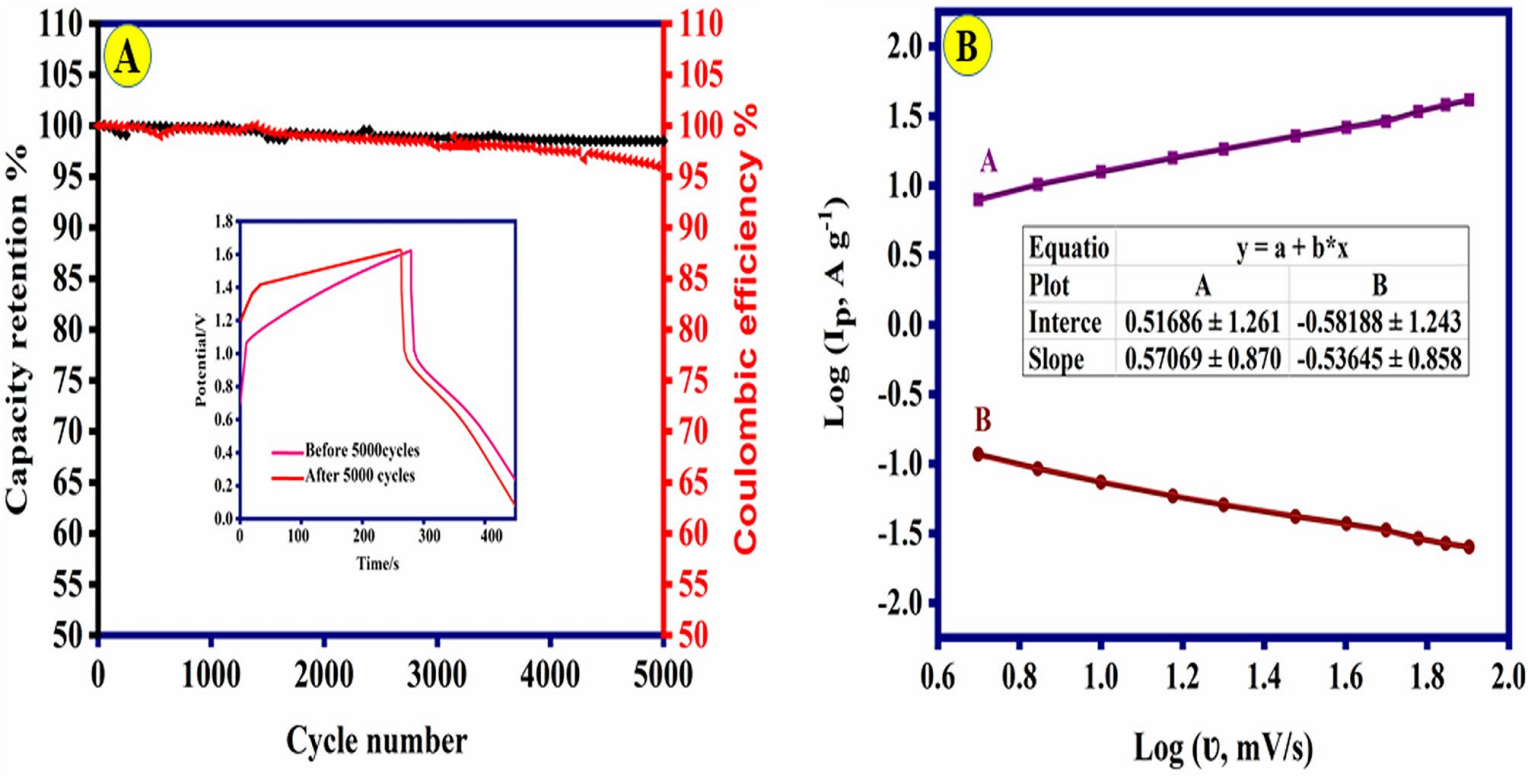
(**A**) Cycling performance of Ni-Co(3:1)@PS-CC electrode at 9.0 A g^−1^ (the inset shows the GCD of the electrode before and after 5000 cycles). (**B**) log I−log ʋ curves of Ni-Co(3:1)@PS-CC electrode (anodic and cathodic peak current).

The results indicate that Ni-Co(3:1)@PS-CC was able to maintain a specific capacitance of 45.81% with an increase in current density from 1.0 to 9 A g^−1^, (Fig. 4F). One of the most prominent features for evaluating supercapacitors is cyclic stability. Figure 5A, illustrates the outstanding stability of the Ni-Co(3:1)@PS-CC electrode after 5000 sequential cycles at a current density of 9.0 A g^−1^. Therefore, 95.9% of the initial specific capacitance is maintained after 5000 cycles. Furthermore, the Coulombic efficiencies (Faradaic efficiencies) are measured as 98.4% after 5000 cycles. The investigation focuses on analyzing the charge storage kinetics to determine if diffusion-controlled behavior or capacitive-dominated processes are present. This analysis examines the logarithmic connection between peak current density and sweep rate, as depicted in Fig. 5B. In theory, the charge-storage mechanism of the material is analyzed between current (i) and scan rate (v) through the following formula^49^:

4 $${\text{I }} = {\text{ a}}\nu^{{\text{b}}}$$

5 $${\text{Log I }} = {\text{ blog}}\left( \nu \right) \, + {\text{ log}}\left( {\text{a}} \right)$$

where a and b are changeable constants. The b-value ranges from 0.5 to 1 and depends on the ratio of capacitive and diffusion-controlled processes. If b = 0.5, Faradaic ion extraction and insertion entirely regulate the redox through diffusion, while 1 indicates totally capacitive control. It concludes that the b value of Ni-Co(3:1)@PS-CC are 0.53 and 0.57 corresponding to anodic and cathodic peak, which further affirms the coexistence of two kinds of charge storage mechanism in these electrochemical processes. Dunn’s method was utilized to categorize the entire capacity into two distinct components: capacitive behavior and diffusion-controlled behavior. Hence, the present reaction can be characterized as the amalgamation of two distinct processes: a capacitive component (k_1_v) and a diffusion-controlled component (k_2_ν^1/2^), both occurring at a constant potential (V).

6 $${\text{i}}\left( \nu \right) \, = {\text{ k}}_{{1}} \nu \, + {\text{ k}}_{{2}} \nu^{{{1}/{2}}}$$

where the k_1_ and k_2_ represent constants. Deform that equation into the following equation:

7 $$\frac{i}{{\nu^{{1/2{ }}} }} = {\text{ k}}_{{1}} \nu^{{{1}/{2}}} + {\text{ k}}_{{2}}$$

**Figure 6:**
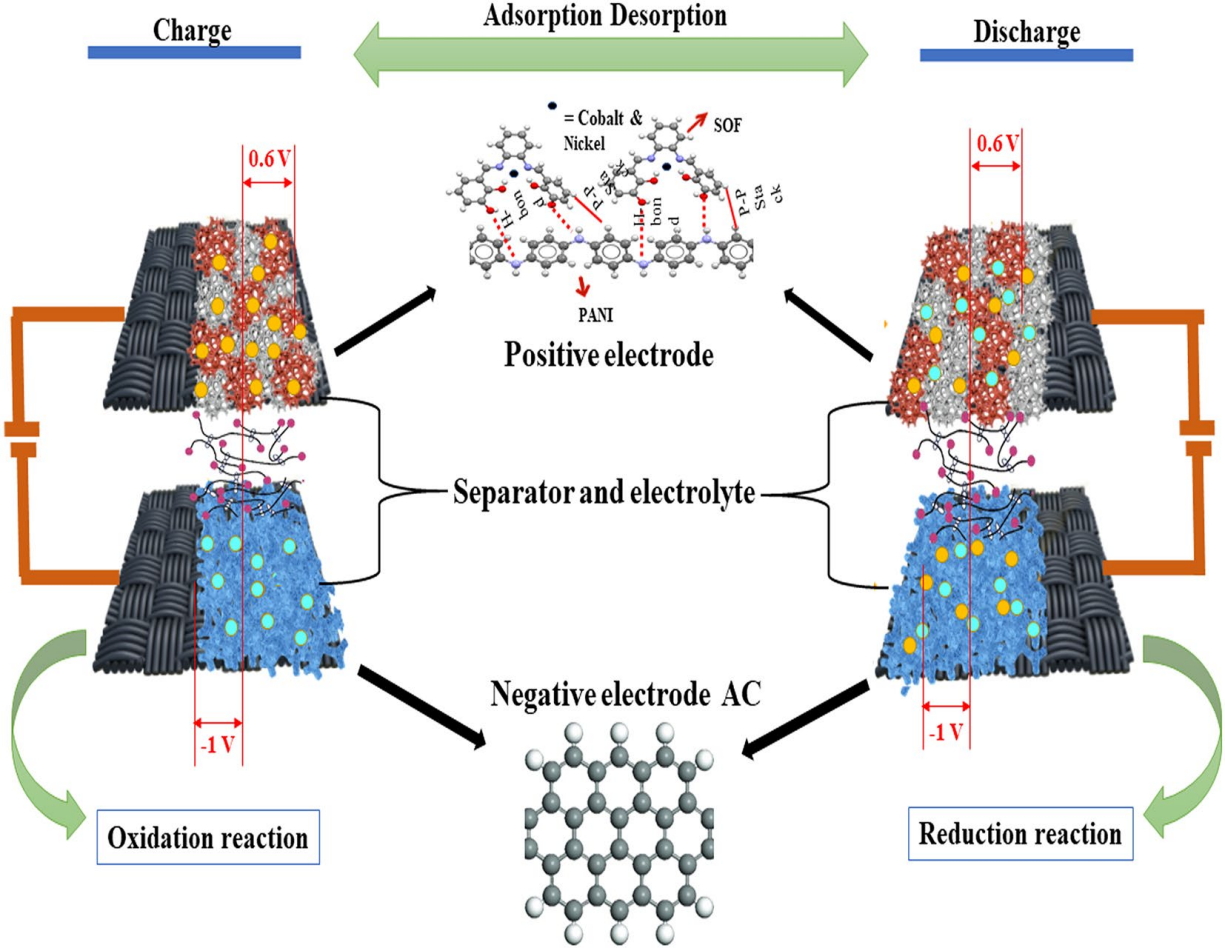
Schematic diagram of the energy storage mechanism.

The values of k_1_ and k_2_ can be determined by analyzing the linear correlation between i/v^1/2^ and ν^1/2^. The values are employed for the assessment of the current arising from both the diffusion-controlled and capacitive-controlled processes. It can be noted that the diffusion-controlled contribution of Ni-Co(3:1)@PS-CC electrode is 75.7% at 5 mV s^−1^ indicating a diffusion-controlled process. The complete energy storage mechanism of SC equipment is shown in Fig. 6.

**Figure 7:**
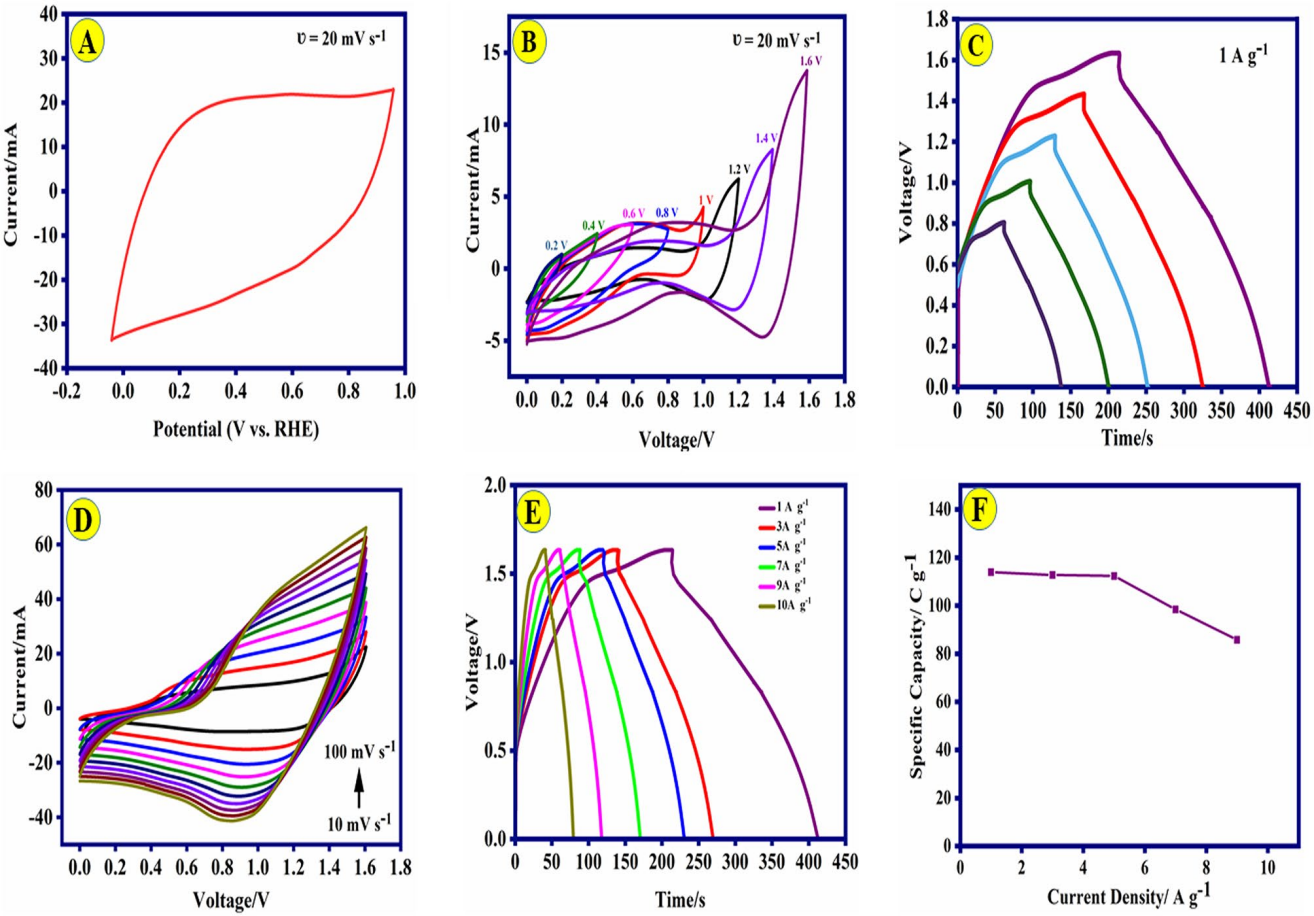
(**A**) Cyclic voltammogram of AC-CC electrode (at the scan rate 20 mV s^−1^). (**B** and **C**) Cyclic voltammogram and charge and discharge curves of Ni-Co(3:1)@PS-CC//AC at various potential windows (0.00–1.60 V); (**D**) cyclic voltammogram of Ni-Co(3:1)@PS-CC//AC. at various scan rates; (**E**) Charge and discharge profiles of Ni-Co(3:1)@PS-CC//AC at various current densities (1.0–100.00 A g^−1^); (**F**) plot of the specific capacitance vs. current density of the Ni-Co(3:1)@PS-CC//AC ASC.

As a further in-depth study, Ni-Co(3:1)@PS-CC electrode was used to fabricate an asymmetric wearable supercapacitor. In this regard, Ni-Co(3:1)@PS-CC and activated carbon@CC (AC-CC) were used as positive and negative electrodes with TCF as separator and PVA/KOH as an electrolyte. Figure 7A illustrated the CV curve of the AC-CC electrode in three-electrode systems at a constant scan rate of 20 mV s^−1^. Correspondingly, Fig. 7B displayed the CV curves of Ni-Co(3:1)@PS-CC//AC at different potential windows with scan rate of 20 mV s^−1^. The significant practical applications of this ASC device are its broad range of operating voltage (Fig. 7B). A potential of 1.60 V was selected for further investigation due to its stability, as opposed to the potential of 1.80 V, which resulted in the occurrence of the oxygen evolution reaction (OER) and subsequently reduced the long-term stability of the device.

Figure 7C exhibited the GCD curves of Ni-Co(3:1)@PS-CC//AC at different potential range and at 1.0 A g^−1^. The asymmetrical nature of the GCD curve observed for Ni-Co(3:1)@PS-CC//AC suggested battery-type behavior, as evidenced by the pro-longed discharging time related to the charging time. Consequently, the geometry of the GCD curves demonstrates notable capacitive properties. CV curves of Ni-Co(3:1)@PS-CC//AC were also obtained at different scan rates (10–100 mV s^−1^) in a voltage window of 0.00–1.60 V (Fig. 7D). A linear relationship between the specific capacitance and scan rate was observed. The absence of distortion and the increase in current density with higher potential scan rates suggest a favorable rate capability for the Ni-Co(3:1)@PS-CC//AC electrode. Figure 7E demonstrates the GCD plots of the Ni-Co(3:1)@PS-CC//AC at different current densities from 1.0 to 10.0 A g^−1^. The specific capacity of Ni-Co(3:1)@PS-CC//AC at 1.0, 3.0, 5.0, 7.0 and 9.0 A g^−1^ was calculated as 113.91, 112.79, 112.34, 98.42 and 85.77 C g^−1^, respectively. After increasing the current density from 1.0 to 9 A g^−1^, 75.29% of the specific capacitance remains, demonstrating its acceptable rate capability (Fig. 7F).

**Figure 8:**
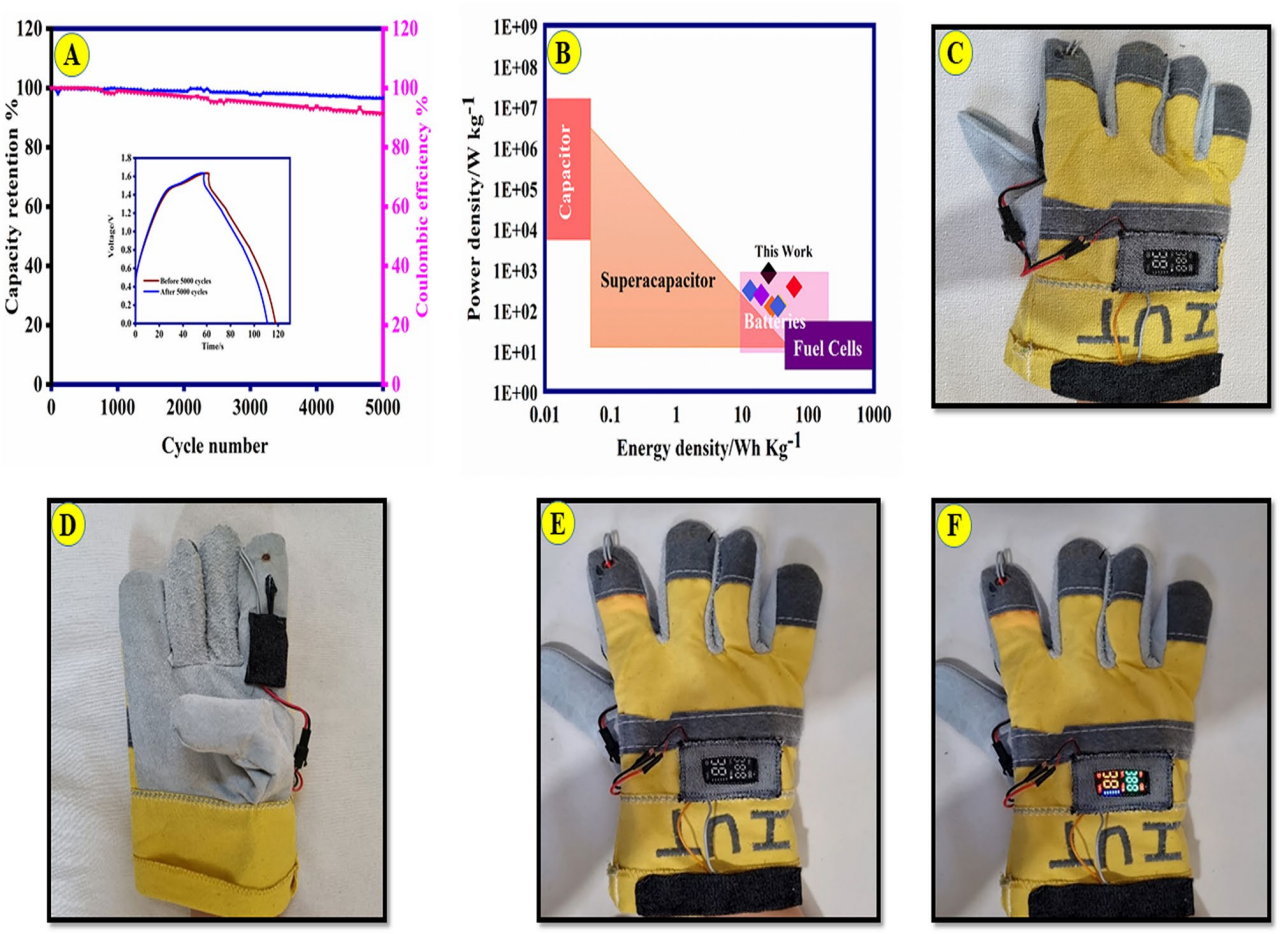
(**A**) Ni-Co(3:1)@PS-CC//AC devise stability test at 9.0 A g^−1^ (the inset indicates the GCD of the primary electrode and after 5000 cycles); (**B**) Ragone plot of Ni-Co(3:1)@PS-CC//AC device; (**C**) the digital image depicts a wearable supercapacitor that has been integrated into a glove for the purpose of powering an oximeter.

Energy density and power density are crucial characteristics for evaluating the effectiveness of an ASC device. Ni-Co(3:1)@PS-CC//AC demonstrates high energy density and power density of 25.2 Wh kg^−1^ and 800 W kg^-1^, respectively. The stability of the wearable supercapacitor is a vital aspect that holds significant importance in various practical applications. The present study demonstrates the sustained stability of Ni-Co(3:1)@PS-CC//AC at a current density of 9 A g^-1^ for a total of 5000 cycles, as illustrated in Fig. 8A. The Ni-Co(3:1)@PS-CC//AC exhibited a capacitance retention rate of approximately 91.3% following 5000 cycles. The composite’s elevated high stability is ascribed to the presence of a stable conducting polymer (PANI) that acts as a binding agent for all constituents and fixes the active material onto the electrode's surface. On the other hand, PANI composite with a lower band gap energy facilitates enhanced charge transport, resulting in superior super-capacitive performance^50^. Furthermore, the unique structure of the salphen complex not only prevents the dissolution of the metal ions but also improves the stability and reusability of the prepared nanocomposite. Furthermore, the Coulombic efficiencies (Faradaic efficiencies) are measured as 96.6% after 5000 cycles Fig. 8A.

Figure 8B presents a summary of the Ragone plots for the wearable supercapacitor using Ni-Co(3:1)@PS-CC//AC composite^50–55^. Compared to other conductive composite, the Co(3:1)@PS-CC//AC supercapacitor has significantly higher energy densities and power densities. As a demonstration in Fig. 8C, a tandem wearable supercapacitor consisting of four individual supercapacitors connected in series was fabricated, resulting in a functional voltage output of 3.5 V. The integrated supercapacitor was then sewn onto the inner layer of a glove and utilized to power a single commercial oximeter. Modifications have been implemented to the commercial oximeter in use, entailing the segregation of the photodetector, light source, and power source. The establishment of the electrical connectivity between the oximeter parts and the wearable supercapacitor was facilitated through the utilization of wire and their subsequent integration into the glove via sewing. Two separate supercapacitors, which are made in parallel, double, and quadruple, were used to turn on the red lamp and board (screen and board), respectively. The all-solid-state wearable supercapacitor was fabricated using functional and sandwich layers made entirely of fabrics, allowing for easy integration of the wearable supercapacitor into the glove through simple sewing techniques. The evaluation of the supercapacitor performance of the Ni-Co(3:1)@PS-CC composite in comparison to previous literature composite-based supercapacitor electrode materials are given in Table S4, evidently demonstrates the exceptional supercapacitor performance.

## Materials and methods

Distillated aniline (Ani), ortho-phenylenediamine (OPDA) and salicylaldehyde, were acquired from Sigma-Aldrich. Hydrochloric acid (HCl), ammonium persulfate (APS), Ni(NO_3_)_2_⋅6H_2_O, Co (NO_3_)_2_.6H_2_O, potassium hydroxide (KOH), graphite powder, activated carbon (AC), carbon black (CB), polyvinylidene difluoride and Ethanol, were purchased from Merck Company (Darmstadt, Germany). Acetone (C_3_H_6_O) was purchased from Mojallali Company (Tehran, Iran). A conductive carbon cloth (CC, 100 × 100 × 1.5 mm) was purchased from Nano-bazaar Company (Tehran, Iran). All other chemicals and reagents utilized were of analytical grade. In all the experiments, deionized water was used throughout the experiment. To avoid the short circuit a piece of thin cotton fabric (TCF) (0.4 mm) was used in AWSC. Surface oxidation and composition of the synthesis material electrode were conducted through X-ray photoelectron spectroscopy (XPS, Bes Tec 8025), Brunauer–Emmett–Teller (BET, Microtrac Bel Corp., Model BElSORP Mi) and morphological analysis and crystal struct of the composite were invested by field-emission scanning electron microscopy (FE-SEM, FEI Quanta FEG 450), and X-ray diffraction (XRD, ASENWARE, AW-XDM300), and coupled plasma-atomic emission spectroscopy measurements (ICP-AES). The FT-IR spectra of the compounds were recorded on a Perkin Elmer (Model Spectrum GX).

Cyclic voltammetry (CV), galvanostatic charge/discharge (GCD), and electrochemical impedance spectroscopy (ESI) were conducted using a Bio-Logic SP300 instrument equipped with a three-electrode system containing (1.0 × 1.0 cm^2^) Ni-Co@PS-salphen-CC (Ni-Co@PS-CC) as a working electrode and Ag/AgCl and Pt wire as a reference and counter electrodes, respectively.

### Synthesis of metal salphen complex

#### First steps

Salphen complex was synthesized by the following procedure described^24^ as follows: phenylenediamine (954 mg, 2.00 mmol) and salicylaldehyde (244 mg, 2.00 mmol) were added to a mixture of methanol (8 mL) and chloroform (8 mL), and the mixture was dispersed by an ultrasonic probe for about 5 min, followed by refluxing at 70 °C. After 2 h, the fine yellow precipitate (salphen) was filtered, and it was filtered and rinsed several times with ethanol. Finally, the obtained yellow powder was dried in a vacuum oven at 50 °C for 24 h.

#### Second steps

Five types of Ni-Co@S containing various percentages of the metals ions (w/w%) were prepared as shown in Table S1. The synthesis process involves the utilization of the values specified within Table S1. First, Co(NO_3_)_2_∙6H_2_O and (Ni(NO_3_)_2_·6H_2_O) were sonicated in ethanol solution (25 mL) for 30 min. Then, the salphen complex powder (1.0 g) was added to the solution. The mixture was sonicated for 15 min and refluxed at 70 °C for 2 h. Finally, the dark brown precipitate was subjected to ethanol washing and subsequently undergone to a vacuum oven at 50 °C for a duration of 24 h^56^.

### Synthesis of Ni/Co@PS

The different types of composite Ni-Co@PS (with different metals percentages, Table S1) were fabricated via in-situ polymerization. Ni-Co@PS composite was synthesized by using two different solutions A and B. First, Ni-Co@S was added into 50 mL HCl (1.0 M) and it was sonicated for 20 min (solution A). Solution B was prepared by combining (4.5 mL) of aniline monomer and 30 mL of HCl solution (1.0 M). Then, solution A was added to solution B under sonicating for 15 min. The above mixture was kept under a magnetic stirrer at 5 °C (ice bath). Afterward, the fresh ammonium persulphate solution (30 mL, 3.0 M), as an oxidant agent, was dropped slowly into the mentioned solution for 30 min while the stirring was maintained for 16 h. Lastly, the obtained green dark powder was dried in a vacuum oven at 50 °C for 24 h.

### Fabrication of wearable symmetric supercapacitor

The wearable electrode was fabricated from commercially available CC (1.0 × 1.0 cm^2^), which was pretreated by HNO_3_ at 80 °C for 10 h to concern hydrophilicity to the surface^57^. The residual acid was eliminated through the application of ethanol, acetone, and deionized water, respectively. Finally, the treated CC was allowed to dry under ambient conditions for further use. The electrode materials were prepared by mixing poly(vinylidene) fluoride (10 wt%) as a binder and the Ni-Co@PS composite (90 wt%) into 2 mL of N-methyl-2-pyrrolidone under stirring and subsequent ultrasonication for 60 min. To fabricate the final electrode, pieces of CC were subjected to the afore-mentioned solution under stirring for 4 h. A series of experiments including seven distinct stirring durations were conducted in order to determine the optimal time as shown in Table S2. Then, the electrode was dried at 50 °C for 12 h to produce Ni-Co@PS-CC^57^. For the asymmetric device, a piece of non-conductive thin cotton fabric (TCF) was utilized to avoid the short circuit. The TCF was sandwiched by one pair of the modified CC, Ni-Co@PS, and active carbon (AC). Here, a solid electrolyte composed of polyvinyl alcohol (PVA)/KOH hydrogel is employed. The solid electrolyte was fabricated by dissolving 3.0 g of PVA monomer and 2.2 g of KOH into 30 mL of water at 90 °C under stirring for 50 min. After preparing the hydrogel, each separator TCF was coated with PVA/KOH hydrogel. To maintain the device's integrity, a thin layer of Whatman filter paper was utilized to wrap around it. Ultimately, the complete apparatus was enveloped with hydrophobic polyester textiles using a hot sealing process and left for 12 h until the hydrogel solidified^9,36^. The proposed synthesis pathway of Ni-Co@PANI-salphen composite was shown in Scheme S1.

## Conclusion

A novel Ni-Co(3:1)@PS-CC composite has been successfully synthesized via in-situ polymerization at room temperature. The Ni-Co(3:1)@PS-CC exhibits elevated doping levels, enhanced charge transfer capacity, improved electrochemical kinetics, and superior energy storage characteristics compared to the other hybrid polymer. The composite structure with hydrogen bond and stacking bond between PANI and metal salphen complex exhibited a high specific capacitance of 549.994 C g^-1^ (1447.2 F g^−1^) at a current density of 0.5 A g^−1^, and a good rate capability and long cyclic stability of this electrode (95.9% recovery after 5000 cycles). Additionally, (FE-SEM, XPS, BET, FT-IR, XRD, …) were carried out to confirm the structure. In addition, the Ni-Co(3:1)@PS-CC//AC asymmetric supercapacitor indicates a specific capacitance, energy density, and power density, 70.01 F g^−1^, 23.46 W h kg^−1^, and 800 W kg^−1^, respectively.

### Supplementary Information

Supplementary Information.

## Data Availability

The datasets supporting the conclusions of this article are included within the article.
