# Supplementary material for: Flexible and sewable electrode based on Ni-Co@PANI-salphen composite-coated on textiles for wearable supercapacitor
Source: Sci Rep. 2023 Nov 13;13:19772. doi: 10.1038/s41598-023-47067-y (PMC10643400; doi:10.1038/s41598-023-47067-y)
Supplement: Supplementary file 1 — Supplementary Information. [file 41598_2023_47067_MOESM1_ESM.docx]

**Supporting information**

**Flexible and** [**sewable**](https://www.sciencedirect.com/science/article/pii/S0378775323004305) **electrode based on Ni-Co@PANI-salphen composite -coated on textiles for wearable supercapacitor**

**Touba Rezaee Adriani^1^, Ali A. Ensafi*^1,2^, B. Rezaei**

*^1^Department of Chemistry, Isfahan University of Technology, Isfahan 84156-83111, Iran.*

*^2^ Adjunct Professor, Department of Chemistry & Biochemistry, University of Arkansas, Fayetteville, AR 72701, USA.*

**Table S1.** Optimizing the substrate percentage in the synthesis of various composites.

| Electrode material | Ni(NO_3_)_2_.6H _2_O (mg) | Co(NO_3_)_2_.6H _2_O (mg) | Salphen (mg) |
| --- | --- | --- | --- |
| Ni-Co | 0.0 | 0.0 | 1000.0 |
| Ni-Co(3:1) | 46.1 | 15.3 | 1000.0 |
| Ni-Co(2:1) | 40.9 | 20.4 | 1000.0 |
| Ni-Co(1:3) | 15.3 | 46.1 | 1000.0 |
| Ni-Co(1:2) | 20.4 | 40.9 | 1000.0 |

**Table S2.** The fabricated Ni-Co(3:1)@PS with different stirring durations.

| time | 30 min | 1 h | 2 h | 3 h | 4 h | 5 h | 5 h 30 min |
| --- | --- | --- | --- | --- | --- | --- | --- |
| Weight (mg cm^-2^) | 0.005 | 0.045 | 0.089 | 0.96 | 1.2 | 1.23 | 1.31 |
| Capacity (C g^-1^) | 212.598 | 336.4 | 440 | 471.25 | 559.996 | 510.345 | 490.217 |

**Table S3.** IR spectral data of salophen and its Ni-Co(1:3)@S, PANI and Ni-Co(3:1)@PS (cm^-1^).

| **Compound** | **υ(HC=N)** | **υ (N-H)** | **υ(C-N)** | **υ(C-H)** | **υ(C=C)** | **υ(M-O)** | **υ(M-N)** | **Ref.** |
| --- | --- | --- | --- | --- | --- | --- | --- | --- |
| complex | 1481.35 | 3071.42 | 1613.12 | - | 1504.70 | - | - | 1,2 |
| Co-Ni@complex | 1477.04  1458.55 | 3417.00 | 1611.04 | - | 1504.60  1559.79 | 524.46 | 439.98 | 1,2 |
| PANI | - | 3432.20 | 1134.07 | 29204.5  2850.00 | 1563.05  1488.69 | - | - | 3 |
| Ni-Co(3:1)@PS | 1458.72 | 3419.00  3063.00 | 1610.20 | - | 1566.00 | 503.00 | 406.42 | 1–3 |

**Table S4.** Electrochemical equation. (A) specific capacitance (Csp, C/g) in a 3-electrode system: l I in terms of A (I, A), specific capacitance (Csp, C/g), potential range (ΔV, V), discharge time(Δt, s), and mass of the active substance coated on the surface (m, g), (B), capacitance (asymmetric) (C, F/g), the mass of the electrode material (m, g) m = m^+^+ m^-^  (C) Electric charges stored (D) The following equation calculated the mass ratio of the solid-state ASCs device (E) coulombic efficiency (η) Δt_d_ (s) represents the discharge time, Δt_c_ represents the charging time, (F) specific energy (E, Wh Kg ^-1^), capacitance (C, F/g), voltage range (ΔV, V), and discharge time (t, s), (G)specific power (P, W Kg ^-1^).

| **A** | **Specific capacity** | **C_s_ =** $\frac{\boldsymbol{ⅰ}\boldsymbol{.\Delta t}}{\boldsymbol{m.\Delta V}}$ |
| --- | --- | --- |
| **B** | **Specific capacity (Asymmetric)** | **C_cell_ =**$\frac{\boldsymbol{ⅰ}\boldsymbol{.\Delta t}}{\boldsymbol{M.\Delta V}}$**, M =m _cathode_ +m _anode_** |
| **C** | **Electric charges stored** | **q =**$\frac{\boldsymbol{C\Delta V}}{\boldsymbol{m}}$ |
| **D** | **Mass ratio** | $\frac{\boldsymbol{m}_{\boldsymbol{+}}}{\boldsymbol{m}_{\boldsymbol{-}}}\boldsymbol{=}\frac{\boldsymbol{C}_{\boldsymbol{-}}\boldsymbol{\Delta}\boldsymbol{V}_{\boldsymbol{-}}}{\boldsymbol{C}_{\boldsymbol{+}}\boldsymbol{\Delta}\boldsymbol{V}_{\boldsymbol{+}}}$ |
| **E** | **coulombic efficiency (η)** | $\boldsymbol{\eta=}\frac{\boldsymbol{\Delta}\boldsymbol{t}_{\boldsymbol{d}}}{\boldsymbol{\Delta}\boldsymbol{t}_{\boldsymbol{c}}}$ |
| **F** | **Energy** | **E =** $\frac{\boldsymbol{C}_{\boldsymbol{cell}}\boldsymbol{.\Delta}\boldsymbol{V}^{\boldsymbol{2}}}{\boldsymbol{7.2}}$ |
| **G** | **Power** | **P =** $\frac{\boldsymbol{E\times3600}}{\boldsymbol{\Delta t}}$ |

**Table S5.** Comparison of some of the supercapacitor performance parameters of the Ni-Co(3:1)@PS with previously reported composite electrode materials.

| **Electrode material** | **Current density** | **Specific capacity (F/g)** | **Capacitance retention % (Cycles)** | **Ref.** |
| --- | --- | --- | --- | --- |
| rGO/PPy | 0.6 mA cm^-2^ | 336.0 | 64.0(500) | 4 |
| Poly[Ni(salphen) | 1.0 mA cm^-2^ | 200.0 F g^-1^ |  | 5 |
| VPPyNTs/CNOs@PPyG) | 0.5 A g^-1^ | 64 .0 F g^-1^ | 50.0(500) | 6 |
| PANI/N-CNT@CNT | 1 A g^-1^ | 323.8 F g^-1^ | 92.1(10000) | 7 |
| Co,N-SnO2/ACF | 1 A g^-1^ | 361.2 F g^-1^ | 103.3(10000) | 8 |
| Ni(CH_3_O-salphen) | 0.05 mA cm^-2^ | 216.0 F g^-1^ | - | 9 |
| Ni-MnO_2_/PANi-co-PPy | 1.0 mA cm^-2^ | 445.49 F g^-1^ | 61.65(5000) | 10 |
| Polyaniline@Mn-TiO_2_ | 1 A g^-1^ | 635.87 F g^-1^ | 91.0(5000) | 11 |
| Zn−In−S/C@CuO | 2 A g^-1^ | 245 C g^-1^ | 100(20000) |  |
| ZrMnoxide@CuO@Cu | 4 A g^-1^ | 550 C g^-1^ | 100(20000) | 12 |
| Ni-Co@PANI-Salphen | 0.5 A g^-1^ | 549.994 C g^-1^ | 94.3(5000) | This work |


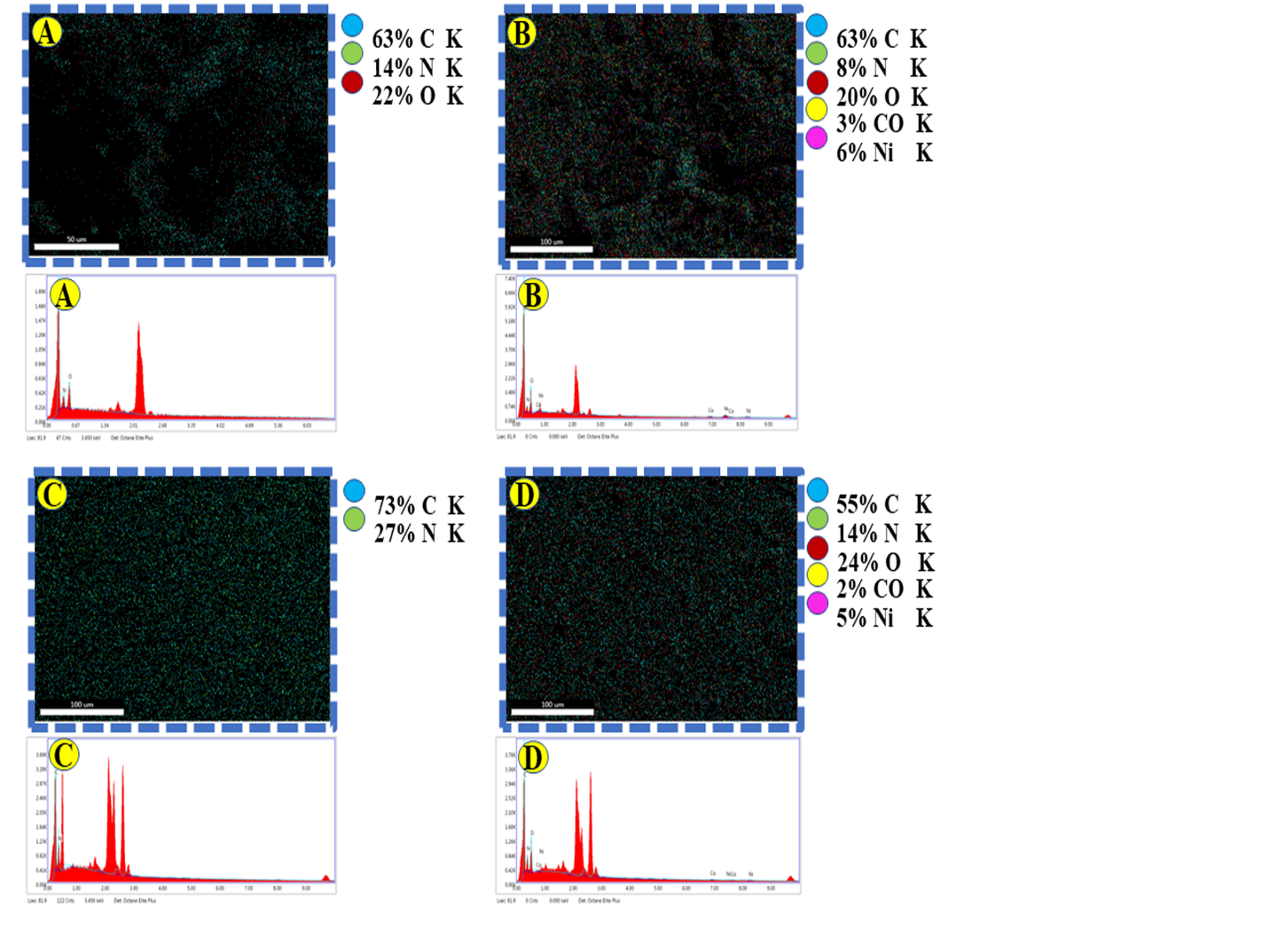


**Fig. S1.** EDX spectrum, elemental mapping of (A) Salphen, (B), Ni-Co(1:3)@S, (C) PANI, (D) Ni-Co(3:1)@PS composite.


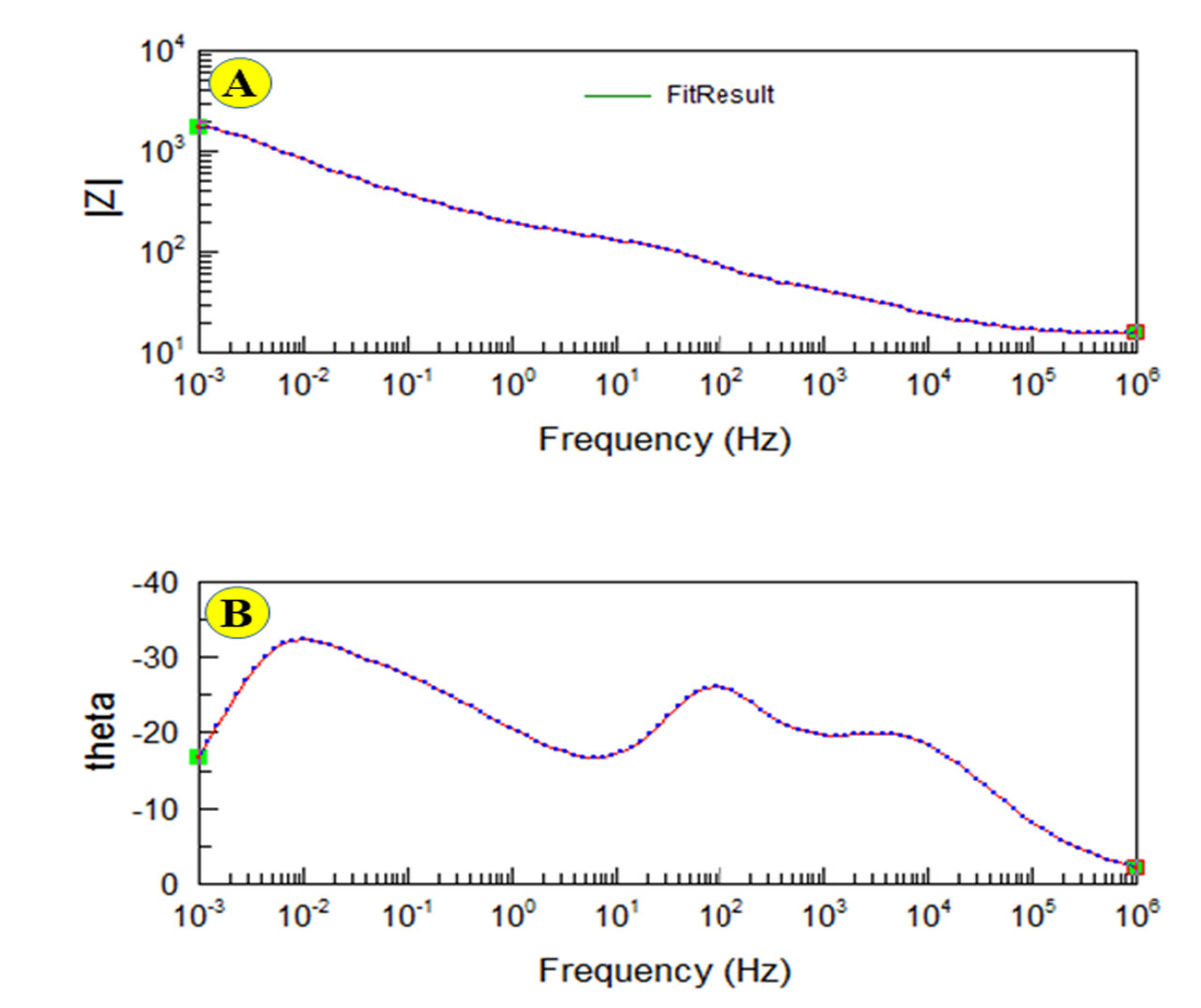


**Fig. S2.** Bode plot of (A) total impedance (|Z|) and (B) phase angle of Ni-Co(3:1)@PS composite


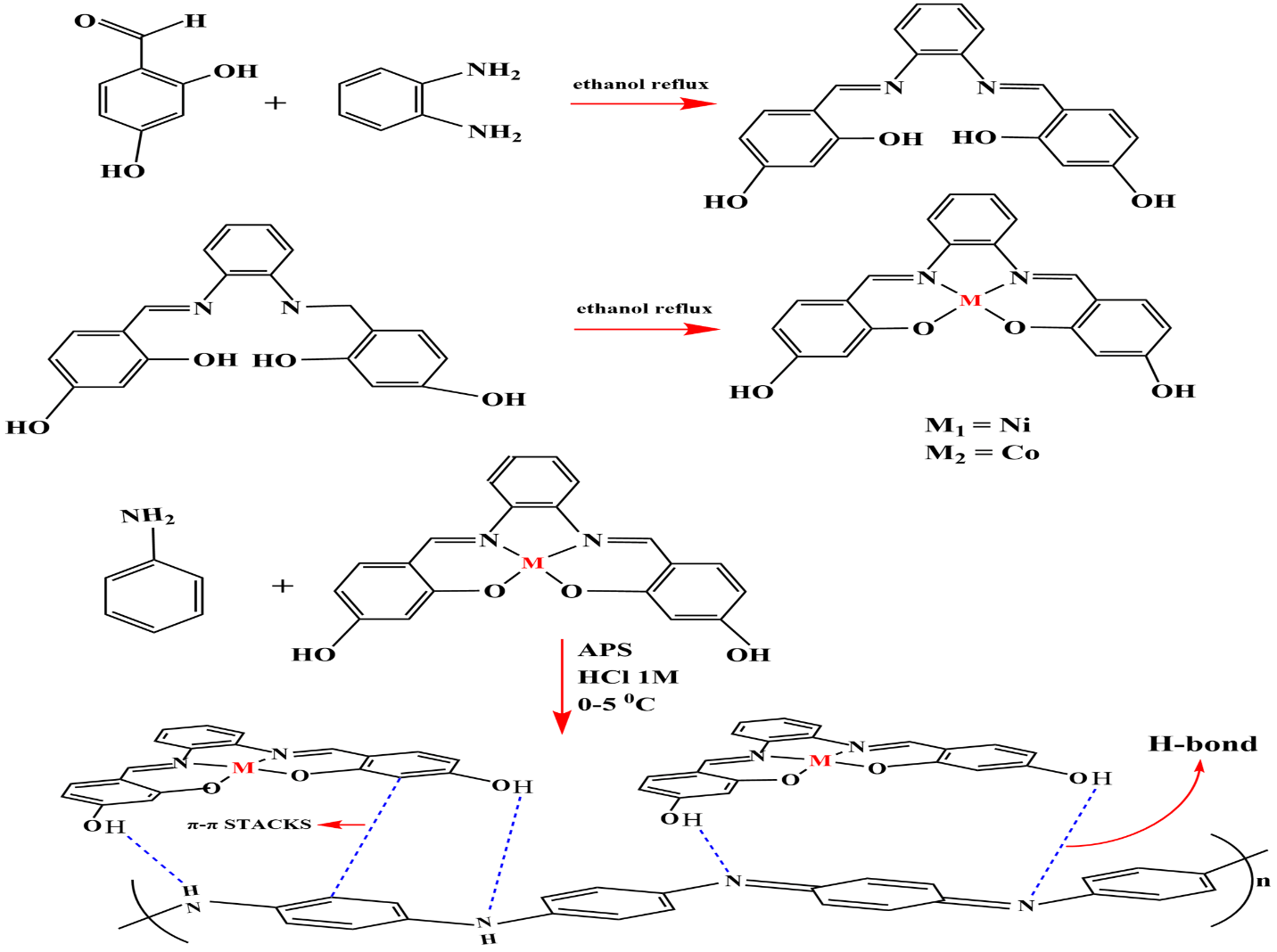


**Scheme S1.** The proposed synthesis pathway of Ni-Co@PANI-salphen composite

**References**

1. Kim, J. H. *et al.* Magnetic metal-complex-conducting copolymer core-shell nanoassemblies for a single-drug anticancer platform. *NPG Asia Mater.* **9**, 3–9 (2017).

2. Ma, W. *et al.* Effects of metal-organic complex Ni(Salen) on thermal decomposition of 1,1-diamino-2,2-dinitroethylene (FOX-7). *RSC Adv.* **10**, 1769–1775 (2020).

3. Pattanayak, P., Papiya, F., Kumar, V., Singh, A. & Kundu, P. P. Performance evaluation of poly(aniline-co-pyrrole) wrapped titanium dioxide nanocomposite as an air-cathode catalyst material for microbial fuel cell. *Mater. Sci. Eng. C* **118**, 111492 (2021).

4. Xu, J. *et al.* Polypyrrole/reduced graphene oxide coated fabric electrodes for supercapacitor application. *Org. Electron.* **24**, 153–159 (2015).

5. Chen, C., Li, X., Deng, F. & Li, J. Electropolymerization and electrochemical behavior of nickel Schiff base complexes with different groups between imine linkages. *RSC Adv.* **6**, 79894–79899 (2016).

6. Wang, L., Zhang, C., Jiao, X. & Yuan, Z. Polypyrrole-based hybrid nanostructures grown on textile for wearable supercapacitors. *Nano Res.* **12**, (2019).

7. Tian, J., Cui, N., Chen, P., Guo, K. & Chen, X. High-performance wearable supercapacitors based on PANI/N-CNT@CNT fiber with a designed hierarchical core-sheath structure. *J. Mater. Chem. A* **9**, 20635–20644 (2021).

8. Xu, J. & Xie, Y. Dual-defects induced band edge reconstruction of tin dioxide via cobalt and nitrogen Co-Doping for wearable supercapacitor application. *J. Power Sources* **493**, 229685 (2021).

9. Deng, F., Li, X., Ding, F., Niu, B. & Li, J. Pseudocapacitive Energy Storage in Schiff Base Polymer with Salphen-Type Ligands. *J. Phys. Chem. C* **122**, 5325–5333 (2018).

10. Dong, J. *et al.* Poly(aniline-co-pyrrole)-coated Ni-doped manganese dioxide as electrode materials for supercapacitors. *Funct. Mater. Lett.* **13**, 1–8 (2020).

11. Poudel, M. B., Yu, C. & Kim, H. J. Synthesis of conducting bifunctional polyaniline@mn-TiO2 nanocomposites for supercapacitor electrode and visible light driven photocatalysis. *Catalysts* **10**, (2020).

12. Hussain, I. *et al.* Theoretical and Experimental Investigation of In Situ Grown MOF-Derived Oriented Zr-Mn-oxide and Solution-Free CuO as Hybrid Electrode for Supercapacitors. *Adv. Funct. Mater.* **33**, 1–10 (2023).
